# Osteocyte Mechanobiology in Peri-Implant Bone Adaptation: A Narrative Review and Hypothesis-Generating Framework for SOST/Wnt-Linked Cortical Stability

**DOI:** 10.3390/cells15090748

**Published:** 2026-04-22

**Authors:** Anna Ewa Kuc, Magdalena Sulewska, Grzegorz Hajduk, Paulina Kuc, Natalia Kuc, Joanna Lis, Beata Kawala, Michał Sarul

**Affiliations:** 1 Department of Dentofacial Orthopedics and Orthodontics, Wroclaw Medical University, 50-425 Wroclaw, Poland; joanna.lis@umw.edu.pl (J.L.); beata.kawala@umw.edu.pl (B.K.); 2 Department of Periodontal and Oral Mucosa Diseases, Medical University of Bialystok, ul. Waszyngtona 13, 15-269 Bialystok, Poland; magdalena.sulewska@umb.edu.pl; 3 Chair and Department of Oral Surgery, Medical University of Lublin, Witolda Chodźki 6 Street, 20-093 Lublin, Poland; hajduk.grzegorz53@gmail.com; 4 Faculty of Medicine, Medical University in Bialystok, ul. Kilińskiego 1, 15-089 Bialystok, Poland; 5 Department of Integrated Dentistry, Wroclaw Medical University, 50-425 Wroclaw, Poland; michal.sarul@umw.edu.pl

**Keywords:** osteocyte, sclerostin, SOST, Wnt signaling, peri-implant bone, bone modeling, marginal bone loss, osseointegration, mechanotransduction, bone microenvironment, osteoimmunology, peri-implant stability, apposition

## Abstract

Peri-implant marginal bone stability remains a major determinant of long-term implant success, yet clinical studies report early marginal bone changes ranging from near-stable conditions in some protocols to approximately 1–2 mm during the first year in more traditional series, underscoring considerable biological variability. In the present review, a remodeling-dominant state refers to turnover-led peri-implant adaptation with limited net cortical gain, whereas modeling-driven apposition refers to uncoupled surface bone addition and cortical reinforcement. We conducted a structured narrative review of PubMed/MEDLINE, Scopus, and Web of Science for literature published between 2000 and February 2026 and qualitatively synthesized direct peri-implant evidence, craniofacial/oral non-implant evidence, and extrapolative mechanobiology from long-bone and systemic models. The available literature supports osteocyte-centered SOST/Wnt regulation as biologically plausible for peri-implant cortical adaptation; however, direct human peri-implant molecular validation remains limited. Based on this synthesis, we propose a hypothesis-generating framework in which mechanical signal profile, microenvironmental stability, and host-related factors influence the probability of transition from a remodeling-dominant to a modeling-dominant peri-implant state. This framework should therefore be interpreted as a testable conceptual model rather than a validated peri-implant mechanism. Its main value lies in organizing current evidence and defining priorities for translational studies that integrate molecular, imaging, and biomechanical endpoints.

## 1. Introduction

Peri-implant marginal bone loss remains one of the principal biological challenges in contemporary implant dentistry. Despite high overall survival rates of dental implants, early and late crestal bone remodeling can compromise long-term stability, aesthetics, and functional outcomes [1,2]. Traditional explanatory models focus predominantly on surgical trauma, inflammatory burden, bacterial colonization, and biomechanical overload [3,4]. However, these paradigms insufficiently explain the marked inter-individual variability in peri-implant bone response observed under comparable clinical conditions.

Bone adaptation is increasingly understood as a tightly regulated process governed not only by osteoblast–osteoclast coupling, but by osteocytes acting as central mechanosensitive regulators [5,6,7]. Osteocytes form an interconnected lacuno-canalicular network capable of sensing mechanical strain and fluid shear stress, translating physical signals into molecular responses [8,9,10]. Among these responses, the expression of sclerostin (SOST), a glycoprotein produced primarily by osteocytes, plays a pivotal role in modulating Wnt/β-catenin signaling, thereby regulating bone formation [11,12,13,14].

Sclerostin acts as a potent inhibitor of Wnt signaling through binding to LRP5/6 receptors, suppressing osteoblast differentiation and activity [12,15,16]. Genetic disorders such as sclerosteosis and van Buchem disease, characterized by reduced SOST activity, demonstrate excessive bone formation, underscoring the central inhibitory function of sclerostin in skeletal homeostasis [17]. Conversely, mechanical unloading increases SOST expression and promotes bone resorption [18], whereas dynamic mechanical loading reduces SOST levels and enhances Wnt signaling [10,19,20]. These findings support the concept of an “osteocyte regulatory framework,” in which local mechanical and microenvironmental cues determine whether bone tissue remains in a remodeling-dominant state or transitions toward modeling-driven apposition.

In the context of dental implants, the peri-implant environment represents a unique biomechanical and biological niche. Unlike natural teeth, implants lack a periodontal ligament and transmit forces directly to surrounding bone [21]. Early healing involves trauma-induced remodeling and the regional acceleratory phenomenon (RAP), characterized by increased turnover rather than stable modeling [22]. While RAP enhances remodeling dynamics, it does not necessarily promote sustained cortical thickening or marginal bone stability. The distinction between remodeling (coupled resorption and formation) and modeling (uncoupled surface apposition) becomes particularly relevant in peri-implant cortical preservation [23].

Emerging evidence suggests that peri-implant bone stability is influenced by microenvironmental factors such as vascularization, inflammatory resolution, and mechanical microstrain distribution [24,25]. However, the osteocyte-centered regulatory axis has not been sufficiently integrated into translational implant strategies. Current regenerative approaches focus primarily on scaffold materials, osteoconductive substitutes, or pharmacological agents [26,27], while limited attention has been directed toward non-pharmacological modulation of the osteocyte signaling network.

Recent reviews have addressed peri-implant bone metabolism, implant osseointegration signaling, and the relevance of sclerostin in oral tissues; however, an explicitly osteocyte-centered synthesis linking mechanotransduction, peri-implant cortical adaptation, and the SOST/Wnt axis remains underdeveloped [28,29,30]. The aim of the present article is therefore not to validate a single peri-implant pathway, but to provide a structured narrative synthesis of the available evidence and to propose a testable conceptual framework for future peri-implant research. The present review therefore uses the broader mechanobiology literature as conceptual background, while the proposed peri-implant framework is treated as hypothesis-generating rather than validated.

## 2. Review Approach and Evidence Framing

This article was conducted as a structured narrative review rather than a systematic review or meta-analysis. PubMed/MEDLINE, Scopus, and Web of Science were searched for literature published between 2000 and February 2026 using combinations of the terms osteocyte, sclerostin, SOST, Wnt, Wnt/β-catenin, mechanotransduction, mechanical loading, peri-implant bone, osseointegration, marginal bone loss, peri-implantitis, cortical bone, inflammation, and angiogenesis. Reference lists of key articles were additionally screened to identify relevant foundational studies.

Studies were selected on the basis of conceptual relevance to osteocyte-mediated regulation of peri-implant bone adaptation. Priority was given to original mechanistic studies, translational implant studies, human biomarker studies, and high-quality reviews that informed the osteocyte/SOST/Wnt axis. To improve evidence transparency, the literature was narratively grouped into three categories: direct peri-implant evidence, craniofacial or oral evidence not specific to implants, and extrapolative evidence from long-bone, systemic, or non-craniofacial models. No formal risk-of-bias scoring was performed because the objective of the review was conceptual synthesis across heterogeneous mechanistic and translational study types.

Throughout the manuscript, the proposed framework is presented as a hypothesis-generating interpretation of convergent evidence rather than a validated clinical mechanism. Overall, the supporting evidence was judged to be low-to-moderate, with stronger support for core osteocyte mechanobiology than for direct human peri-implant molecular validation.

## 3. Osteocyte as the Central Regulator of Bone Adaptation

Most mechanotransduction evidence discussed in this section derives from long-bone, calvarial, or in vitro systems rather than from human craniofacial peri-implant bone. Accordingly, these studies are used here as mechanistic background supporting biological plausibility, not as direct peri-implant proof.

Bone is no longer understood as a tissue regulated solely by the dynamic balance between osteoclast-mediated resorption and osteoblast-driven formation. Instead, the osteocyte has emerged as the principal orchestrator of skeletal adaptation, integrating mechanical, metabolic, and inflammatory signals into coordinated molecular responses [31,32]. Embedded within the mineralized matrix, osteocytes form an extensive lacuno-canalicular network that enables mechanosensation through fluid shear stress and deformation-induced strain amplification [33].

Mechanical loading generates interstitial fluid flow within the canalicular system, which activates intracellular signaling pathways involving calcium flux, nitric oxide production, prostaglandins, and downstream transcriptional responses [18,34]. One of the most important consequences of this mechanotransduction cascade is modulation of sclerostin (SOST) expression [35]. In experimental mechanobiology, dynamic loading generally refers to cyclic or oscillatory stimulation within physiologic frequency ranges, commonly around 0.5–2 Hz, with osteocyte responses reported across strain magnitudes on the order of approximately 250–3000 μɛ in vivo and related experimental models. By contrast, static or near-static loading lacks temporal variability and is typically associated with signal attenuation and weaker anabolic signaling. Craniofacial peri-implant loading thresholds, however, remain insufficiently defined and should not be assumed to mirror long-bone values directly [36,37].

Beyond mechanical input, osteocyte behavior is influenced by the local microenvironment. Hypoxia, inflammatory cytokines, and vascular supply modulate SOST expression and Wnt signaling activity [38,39,40,41]. Chronic inflammatory states may maintain osteocytes in a SOST-high, remodeling-dominant state, favoring resorption over modeling. In contrast, a well-perfused and inflammation-resolving environment may support SOST suppression and cortical apposition. Within a broader mechanobiological context, this switching behavior may represent one manifestation of threshold-dependent regulation observed across multiple craniofacial tissues.

Age represents another critical determinant of osteocyte regulatory capacity. Increased sclerostin levels and reduced mechanosensitivity have been reported with aging, consistent with diminished periosteal modeling potential in older bone. This age-related shift suggests that adult peri-implant bone may require stronger or more favorable local cues to achieve net cortical apposition. Direct peri-implant age-stratified molecular evidence, however, remains limited [42,43].

Collectively, these findings support the concept of an osteocyte regulatory axis acting as a molecular framework between remodeling-dominant and modeling-dominant states. Within this framework, SOST serves as a central inhibitory gatekeeper of osteogenesis. The ability to modulate this osteocyte state should therefore be regarded as a hypothesis-generating research direction rather than an established peri-implant therapeutic pathway.

## 4. Peri-Implant Bone: Remodeling Versus Modeling Dynamics

Peri-implant bone healing has traditionally been described through the lens of osseointegration, a process characterized by direct bone-to-implant contact without interposed soft tissue [21,44]. Early healing following implant placement is dominated by trauma-induced remodeling, involving inflammatory signaling, osteoclast activation, and subsequent osteoblast-mediated bone formation [22]. This regional acceleratory phenomenon (RAP) enhances turnover and facilitates structural adaptation; however, increased turnover does not necessarily equate to stable cortical thickening or long-term marginal preservation [45].

A fundamental distinction must therefore be made between bone remodeling and bone modeling. Remodeling refers to coupled resorption and formation occurring at the same site, primarily maintaining structural integrity and repairing microdamage [23,46]. In contrast, modeling involves uncoupled surface apposition or resorption, altering bone geometry and thickness [47]. Periosteal modeling, in particular, is responsible for increases in cortical thickness and adaptation to mechanical demands during growth and functional loading [44,48].

In peri-implant contexts, the dominant biological response after surgery is remodeling-driven. Osteoclast-mediated resorption removes necrotic bone resulting from surgical trauma, followed by secondary osteogenesis that establishes osseointegration [22,42,49]. While this sequence is essential for implant stability, it does not inherently promote sustained cortical apposition at the marginal crest. Indeed, early crestal bone loss observed around implants may reflect a remodeling-dominant environment rather than a modeling-dominant one [50].

The absence of a periodontal ligament fundamentally alters load transmission. Natural teeth distribute occlusal forces through a viscoelastic PDL, whereas implants transmit forces directly to surrounding cortical bone [51]. This direct transmission modifies local strain distribution and may affect osteocyte mechanosensitivity thresholds. This threshold-dependent regulatory behavior aligns with broader mechanobiological concepts in which tissue outcomes emerge from interactions between mechanical input, microenvironmental stability, and immune–redox regulation. Finite element studies demonstrate that peri-implant stress concentrations are highest in the crestal cortical region, precisely where marginal bone loss is most frequently observed [24]. However, stress magnitude alone does not determine biological outcome; rather, the nature of strain (static vs. dynamic), microvascular status, and inflammatory resolution influence whether bone transitions toward modeling or remains remodeling-dominant [40,52].

Microenvironmental stability appears particularly critical during early implant healing. Adequate angiogenesis, oxygenation, and resolution of inflammation support osteoblast recruitment and matrix deposition [41]. Conversely, persistent low-grade inflammation or micro-hypoxia may sustain catabolic signaling pathways, indirectly maintaining elevated SOST expression and limiting cortical thickening [10,53]. These observations suggest that peri-implant marginal bone behavior is not purely a mechanical overload problem, but a failure to transition from an early remodeling state to a stabilized modeling state.

Importantly, cortical thickening in adult bone remains biologically possible under specific conditions. Periosteal apposition has been observed in response to functional loading, enthesis adaptation, and certain controlled mechanical environments [47,48]. The limiting factor appears not to be the intrinsic incapacity of adult bone to form new cortex, but rather the absence of appropriate regulatory cues capable of suppressing inhibitory pathways such as SOST and activating Wnt/β-catenin-mediated osteogenesis. The pharmacological evidence discussed below is included only as proof-of-axis relevance, not as support for immediate clinical anti-sclerostin use in implant dentistry. Experimental evidence demonstrates that pharmacological suppression of sclerostin reactivates quiescent bone lining cells and promotes net bone formation, supporting the concept that adult cortical bone retains anabolic responsiveness under appropriate regulatory conditions [37]. Moreover, large randomized clinical trials targeting sclerostin inhibition have demonstrated significant increases in cortical bone mass, thickness, and structural strength, confirming the translational relevance of SOST modulation in humans [54].

Taken together, peri-implant bone dynamics can be conceptualized as a competition between two states: a remodeling-dominant environment characterized by high turnover and marginal instability, and a modeling-dominant environment characterized by net cortical apposition. The transition between these states is likely governed by osteocyte-mediated signaling thresholds. Understanding and modulating this transition represents a critical translational opportunity in implant dentistry.

Direct peri-implant evidence relevant to this interpretation remains limited but is not absent. Human histologic studies have reported higher osteocyte density around immediately loaded implants and associations between osteocyte density and bone-to-implant contact [55,56], while peri-implant crevicular fluid studies have reported increased sclerostin levels in peri-implantitis relative to peri-implant health [57]. In addition, an animal impact-loading model demonstrated transient upregulation of sclerostin and RANKL with reciprocal suppression of β-catenin during peri-implant bone damage and remodeling [58]. Taken together, these findings support biologic plausibility for a remodeling-dominant peri-implant environment, but they do not yet establish a validated causal mechanism for marginal bone loss.

## 5. Proposed Osteocyte-Centered Framework of Peri-Implant Bone Adaptation

**Figure 1:**
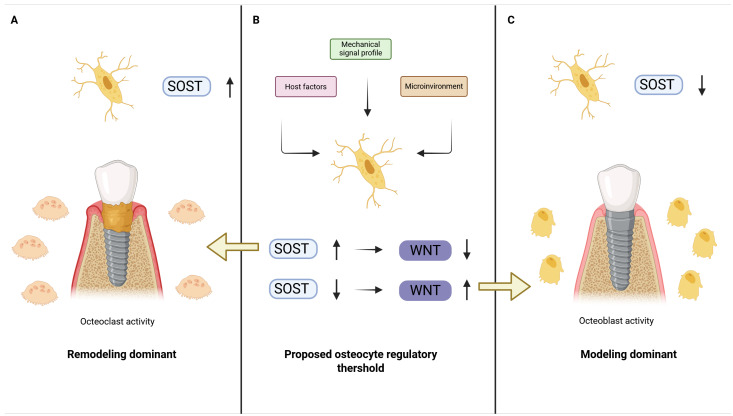
Proposed Osteocyte-Centered Regulatory Continuum of Peri-Implant Bone Adaptation. (**A**) Remodeling-dominant state following implant placement, characterized by increased bone turnover and elevated osteoclast activity. In this state, osteocyte-derived sclerostin (SOST) expression remains relatively high, resulting in suppression of Wnt signaling and maintenance of a remodeling equilibrium without net cortical thickening. (**B**) Regulatory continuum mechanism centered on the osteocyte. Mechanical signal profile (dynamic vs. static loading), microenvironmental stability (perfusion and inflammatory resolution), and host-related biological factors (e.g., age, baseline cortical thickness) converge on the osteocyte regulatory threshold. The balance between SOST expression and Wnt activation determines the transition between remodeling- and modeling-dominant states. (**C**) Modeling-dominant cortical response. Reduced SOST expression and enhanced Wnt signaling favor osteoblast-mediated surface apposition and cortical reinforcement around the implant, potentially contributing to improved marginal bone stability. Illustrative, not empirically calibrated.

Current models of peri-implant bone behavior largely rely on inflammatory and mechanical paradigms, yet fail to fully explain the substantial variability in marginal bone stability observed under apparently similar clinical conditions [25,59]. The present review synthesizes existing biological and biomechanical evidence to outline a proposed osteocyte-centered framework for peri-implant cortical bone adaptation (Figure 1). This framework should be interpreted as a hypothesis-generating model built from convergent but heterogeneous evidence. Overall, the evidentiary support is low-to-moderate: strong for core osteocyte mechanobiology, moderate for implant-specific preclinical and biomechanical inference, and limited for direct human peri-implant molecular validation. The evidence domains underpinning this framework, together with their peri-implant relevance and current limitations, are summarized in Table 1.

This framework does not challenge established principles of osseointegration or remodeling. Rather, it introduces an additional regulatory layer: the transition between a remodeling-dominant and a modeling-dominant cortical state is determined by the molecular setpoint of osteocyte SOST expression and downstream Wnt activity.

Under baseline conditions following implant placement, peri-implant bone undergoes trauma-induced remodeling. Elevated turnover, inflammatory signaling, and mechanical perturbation characterize this early phase [25,44,48,60]. In such a context, osteocyte signaling is likely dominated by adaptive remodeling cues rather than sustained anabolic modeling. Even when bone–implant contact is successfully established, this does not necessarily imply long-term cortical thickening or resistance to marginal bone loss.

The proposed regulatory framework is based on three interacting determinants:

1. Mechanical Signal Profile—Not merely force magnitude, but dynamic characteristics of strain and fluid shear stress within the cortical network [19,24,60,61]. Static or monotonous loading patterns may fail to suppress SOST effectively, whereas cyclic microstrain can induce sustained downregulation [36].
2. Microenvironmental Stability—Adequate perfusion and resolution of inflammation are necessary for Wnt-mediated osteogenesis to dominate [25,39,50,52]. Chronic microinflammatory states may maintain elevated SOST levels and favor resorptive equilibrium.
3. Biological Threshold Factors—Biological threshold factors such as age and baseline skeletal phenotype may influence osteocyte responsiveness [42,43]. These factors modulate the activation threshold required to transition from remodeling to modeling.

These determinants should not be viewed as independent or equally weighted variables. Favorable mechanics may be insufficient when inflammation, hypoxia, or early-healing instability persist, whereas a biologically favorable host state may still fail to produce net cortical apposition if the local mechanical signal remains subthreshold or adverse. The proposed model therefore describes a probabilistic regulatory continuum rather than a binary on/off event.

Within this model, SOST expression acts as a molecular gatekeeper. When SOST remains elevated, peri-implant bone remains in a remodeling-dominant equilibrium, characterized by turnover without net cortical gain. When SOST is sufficiently suppressed—through appropriate mechanical and microenvironmental conditions—Wnt signaling predominates, enabling osteoblast-driven surface apposition and cortical reinforcement.

Importantly, this framework does not imply binary behavior. Rather, it represents a probabilistic regulatory window. The peri-implant cortex exists along a continuum between catabolic-dominant and anabolic-dominant states. Clinical marginal bone loss may therefore reflect a failure to cross the osteocyte activation threshold rather than excessive overload alone.

This framework also explains why trauma-based strategies such as corticotomy or surgical stimulation increase turnover but do not consistently produce stable cortical thickening. Increased remodeling alone does not guarantee transition into a modeling-dominant state unless SOST-mediated inhibition is concurrently reduced.

This framework does not yet provide a mechanistic explanation proven in humans. Rather, it offers a structured, testable interpretation of why peri-implant cortical responses may diverge under apparently similar clinical conditions. By positioning osteocytes—and specifically the SOST/Wnt balance—at the center of the discussion, the model organizes indirect evidence from skeletal mechanobiology, implant biomechanics, and peri-implant healing into a framework for translational testing.

The present review should therefore be regarded as among the first to explicitly frame peri-implant cortical variability through an osteocyte-centered SOST/Wnt lens, rather than as the first conceptual integration of these biological principles. Direct clinical correlations between peri-implant SOST expression and marginal bone loss remain limited, and the framework should be interpreted as a biologically grounded hypothesis requiring further experimental and clinical validation.

## 6. Candidate Non-Pharmacological Research Directions Relevant to the Osteocyte Axis

This section discusses candidate non-pharmacological directions as translational research concepts rather than clinically validated peri-implant interventions. Among them, three appear most immediately testable: staged controlled dynamic loading, load-distribution optimization through implant and prosthetic design, and early-healing microenvironment stabilization. Pharmacological anti-sclerostin studies are considered here only as proof-of-axis relevance, not as direct support for chairside peri-implant therapy [16,37,62]. Candidate non-pharmacological directions relevant to the osteocyte axis are summarized in Table 2.

Mechanical signaling remains the most physiologically relevant regulator of osteocyte activity. For future experimental testing, the most defensible starting point is low-amplitude cyclic stimulation delivered within a physiologic frequency window of approximately 0.5–2 Hz, introduced only after adequate primary stability has been achieved and compared against unloaded, static, and overload conditions. Because implant geometry and bone quality vary substantially, studies should prioritize reporting local strain, micromotion, and stability stage rather than raw force magnitude alone [19,63]. The purpose of such protocols would not be to intensify turnover indiscriminately, but to determine whether defined dynamic loading profiles can reduce SOST and favor net cortical apposition [33,40].

Beyond pure mechanics, the peri-implant microenvironment itself may represent a modifiable determinant of osteocyte state. In peri-implant terms, microenvironmental stabilization may include atraumatic drilling with thermal control, preservation of clot stability and soft-tissue sealing, implant surface or matrix properties that support early angiogenesis, and local biomaterial features that limit persistent inflammatory signaling while maintaining osteoconductivity [25,27,44]. These measures are relevant because osteocyte-mediated anabolic responses are unlikely to dominate in a poorly perfused or persistently inflamed environment.

It is critical, however, to distinguish between increasing bone turnover and inducing net cortical apposition. Interventions that amplify remodeling (e.g., surgical stimulation alone) do not guarantee sustained suppression of SOST or durable modeling [45]. The objective is not to intensify biological activity indiscriminately, but to bias the regulatory balance toward an anabolic setpoint.

Within this conceptual framework, three translational research directions emerge: (1) characterization of mechanical signal profiles capable of safely reducing SOST expression in cortical peri-implant bone; (2) identification of microenvironmental conditions that lower the activation threshold for Wnt-mediated osteogenesis; and (3) development of localized, non-systemic adjunctive technologies designed to transiently modulate osteocyte signaling during the early healing window.

Importantly, such approaches must be validated against objective structural endpoints, including cortical thickness, bone-to-implant contact, and marginal bone stability, rather than relying solely on surrogate molecular markers. SOST suppression alone is insufficient as a therapeutic target unless accompanied by demonstrable improvements in peri-implant structural integrity [62].

The osteocyte regulatory framework therefore does not advocate a single technology, but rather establishes a biological criterion for evaluating future interventions: successful strategies must demonstrate controlled, localized, and reversible modulation of the SOST/Wnt axis leading to net cortical apposition without exacerbating inflammatory or resorptive pathways.

From an experimental standpoint, future validation of the proposed framework should include combined molecular and structural endpoints. Candidate interventions should be evaluated not only for their capacity to modulate SOST expression, but also for their impact on cortical thickness, bone-to-implant contact, marginal bone levels, and biomechanical stability parameters such as resonance frequency analysis. Such integrative assessment is essential to distinguish increased turnover from true modeling-driven cortical reinforcement.

By defining these mechanistic boundaries, the present model provides a structured roadmap for translational development in implant dentistry—grounded in osteocyte biology, constrained by physiological principles, and open to innovation within non-pharmacological domains.

## 7. Clinical Translation, Risk Stratification, and Research Roadmap

The conceptualization of peri-implant bone behavior through an osteocyte-centered regulatory framework carries several important translational implications. First, it reframes marginal bone stability not as a purely mechanical overload problem, nor exclusively as an inflammatory phenomenon, but as a regulatory imbalance within the osteocyte signaling network. This distinction is critical, as it shifts the therapeutic objective from simply minimizing trauma or inflammation toward modulating the molecular thresholds governing cortical modeling.

From a clinical standpoint, the osteocyte regulatory framework model suggests that peri-implant marginal bone loss may reflect a failure to transition from early remodeling to sustained modeling. Current clinical protocols primarily address surgical technique, implant design, and load management [4,24,64]. While these factors remain essential, they do not directly target the intracellular signaling pathways determining whether cortical bone enters a net appositional state. Integrating osteocyte biology into implant treatment planning introduces a new biological dimension that complements, rather than replaces, established biomechanical principles. Consistent with this concept, experimental and clinical studies targeting sclerostin demonstrate that transient suppression of SOST can reactivate quiescent bone lining cells and increase cortical thickness [16,37], providing proof-of-concept for peri-implant anabolic modulation.

Translational development should therefore focus on identifying localized, reversible, and non-systemic strategies capable of modulating SOST/Wnt signaling within the peri-implant cortical compartment. Such strategies must satisfy several criteria: (1) spatial selectivity, to avoid systemic skeletal effects; (2) temporal control, limited to the early healing window; and (3) compatibility with standard implant protocols. The proposed regulatory framework provides a structured benchmark against which emerging technologies—whether mechanical, bioelectrical, or microenvironmental—can be evaluated.

Importantly, clinical translation requires rigorous structural endpoints. Demonstrating reduced SOST expression alone is insufficient without concomitant evidence of increased cortical thickness, improved bone-to-implant contact, or enhanced marginal stability [54,62]. Future investigations must therefore integrate molecular markers with imaging modalities such as micro-CT, CBCT, and resonance frequency analysis to establish clinically meaningful outcomes. Testable predictions and corresponding translational endpoints emerging from the proposed framework are outlined in Table 3.

Three studies appear most urgent for validating the proposed framework. First, a controlled preclinical implant model should combine defined loading regimens with serial readouts of SOST, β-catenin, RANKL/OPG, and structural micro-CT or histomorphometric endpoints. Second, a prospective human cohort study should longitudinally integrate peri-implant crevicular fluid biomarkers, radiographic marginal bone change, and implant stability measurements from placement through at least 12 months of function. Third, a translational pilot intervention study should test one prioritized non-pharmacological strategy—preferably staged loading or early-healing microenvironment optimization—using both biological and structural outcomes.

For future clinical studies, patients could be stratified according to a proposed osteocyte-threshold risk profile. A lower-risk profile would include favorable bone phenotype, good primary stability, absence of smoking, and well-controlled systemic status. An intermediate-risk profile would include a history of periodontitis, posterior maxillary placement, or borderline primary stability. A higher-risk profile would include current smoking, uncontrolled diabetes, repeated early inflammatory complications, poor bone quality, or persistently low stability metrics. This scheme is proposed for study design and hypothesis testing rather than as a validated clinical scoring system.

Peri-implant bone response is further influenced by systemic and patient-related factors that may alter osteocyte regulatory thresholds. Emerging evidence highlights that systemic conditions and local microenvironmental factors affect osteocyte signaling, including SOST/Wnt dynamics, thereby influencing net cortical apposition [65]. Conditions such as diabetes mellitus, smoking, osteoporosis, and medications affecting bone metabolism (e.g., antiresorptives or corticosteroids) can modify SOST expression and Wnt signaling, potentially shifting the osteocyte regulatory setpoint toward a remodeling-dominant equilibrium and reducing the likelihood of transition into a modeling-dominant state. Future translational research should therefore take patient-specific biological context into account when evaluating strategies aimed at modulating peri-implant cortical stability.

The osteocyte regulatory framework model also has implications for personalized implant therapy. This inter-individual variability in mechanosensitivity aligns with the mechanostat concept and strain-dependent regulation of SOST [19,66], underscoring the need for patient-specific modulation strategies. Inter-individual variability in cortical thickness, age-related SOST expression, and mechanosensitivity may explain heterogeneous responses to identical implant designs and loading conditions [45]. A deeper understanding of these regulatory thresholds may enable risk stratification and targeted adjunctive interventions tailored to specific biological phenotypes.

Finally, this framework underscores a broader conceptual shift in implant dentistry: from passive integration to controlled biological activation. Rather than relying solely on osteoconductive scaffolds or surgical modifications, future strategies may aim to transiently bias peri-implant bone toward an anabolic modeling state through controlled modulation of osteocyte signaling. Such an approach aligns with precision medicine principles and opens avenues for innovative, non-pharmacological adjuncts designed to enhance cortical stability without systemic exposure. Clinical trials with anti-sclerostin antibodies (FRAME, ARCH) have demonstrated increases in cortical thickness, supporting the feasibility of targeted SOST modulation for anabolic outcomes [54,62].

Before clinical translation, any peri-implant intervention aimed at the osteocyte axis would need to demonstrate local selectivity, compatibility with standard implant workflows, reproducible timing and dosing, safety for both bone and soft tissues, and measurable benefit in structural peri-implant outcomes. At present, these prerequisites remain unmet, which is why the present review frames such approaches as research directions rather than clinical recommendations.

In summary, the Peri-Implant Osteocyte Regulatory Framework provides a biologically coherent, mechanistically grounded, and translationally actionable model. By situating osteocyte-mediated SOST/Wnt balance at the center of peri-implant regulation, it establishes a foundation for next-generation strategies aimed at improving long-term marginal bone preservation. Understanding osteocyte-mediated regulatory thresholds may help explain inter-individual variability in peri-implant marginal bone stability observed in clinical practice.

## 8. Limitations

Several limitations should be acknowledged. First, this article is a narrative review and conceptual synthesis, not a systematic review; accordingly, study selection and interpretation were not based on formal risk-of-bias assessment or quantitative meta-analysis. Second, the proposed framework integrates evidence across different species, skeletal sites, and experimental conditions, including data not specific to human peri-implant cortical bone. Third, direct peri-implant evidence relevant to SOST/Wnt signaling remains limited and consists largely of preclinical studies, human biomarker observations, and implant histology, which restrict causal inference. Fourth, candidate non-pharmacological modulators of the proposed axis have not yet been validated as selective clinical tools for peri-implant cortical activation. Therefore, the framework should be considered hypothesis-generating and intended to guide future translational and clinical research.

## 9. Conclusions

This narrative review supports the biological plausibility of an osteocyte-centered SOST/Wnt-linked contribution to peri-implant bone adaptation. We propose a hypothesis-generating framework in which mechanical signal profile, microenvironmental stability, and host-related factors influence the probability of transition from remodeling-dominant turnover to modeling-dominant cortical apposition. Because direct human peri-implant molecular validation remains limited, the framework should be interpreted cautiously and tested through studies integrating local biomarkers, structural imaging, and biomechanical endpoints. Its principal value lies in organizing current evidence and defining experimentally tractable questions for future peri-implant research.

## Figures and Tables

**Table 1:** Evidence domains underpinning the proposed osteocyte-centered framework of peri-implant bone adaptation.

Domain	Main Evidence Synthesized in this Review	Direct Relevance to Peri-Implant Bone	Contribution to the Proposed Framework	Main Limitation
Osteocyte mechanotransduction	Osteocytes sense dynamic strain and fluid shear stress and translate mechanical input into molecular responses; cyclic loading is associated with reduced SOST expression and increased anabolic signaling.	Indirect-to-moderate; evidence is strong in general skeletal biology but limited in implant-specific models.	Provides the mechanistic basis for a load-dependent osteocyte response threshold.	Most evidence derives from non-craniofacial and non-implant models.
SOST/Wnt axis	Sclerostin inhibits LRP5/6-mediated canonical Wnt signaling, thereby constraining osteoblast activity and surface apposition.	Indirect-to-moderate; biologically plausible for peri-implant cortex, but direct human peri-implant data remain scarce.	Supports the interpretation of SOST as a molecular gatekeeper between remodeling-dominant and modeling-dominant states.	Few longitudinal studies directly relate peri-implant SOST dynamics to structural outcomes.
Remodeling versus modeling biology	Remodeling repairs trauma through coupled resorption and formation, whereas modeling enables net surface apposition and changes in cortical geometry.	Moderate; directly relevant to interpreting marginal bone behavior after implant placement.	Justifies the distinction between turnover-dominant healing and net cortical reinforcement.	Adult peri-implant crestal modeling has been insufficiently characterized.
Implant-specific biomechanics	Implants lack a periodontal ligament and transmit load directly to surrounding bone; crestal cortical regions experience stress concentration.	Moderate; supported by implant biomechanics and finite element literature.	Explains why similar clinical loading conditions may still produce different local osteocyte responses.	Biomechanical models do not directly measure osteocyte state in vivo.
Microenvironmental stability	Perfusion, oxygenation, inflammatory resolution, and angiogenesis influence bone healing and may modulate osteocyte signaling.	Limited-to-moderate; relevant to early peri-implant healing.	Defines the non-mechanical arm of the proposed threshold model.	Direct peri-implant SOST/Wnt measurements under different microenvironmental states are limited.
Host-related modifiers	Age, systemic disease, smoking, medications, and baseline cortical phenotype may alter mechanosensitivity and osteogenic responsiveness.	Indirect; clinically plausible but not yet well stratified in peri-implant studies.	Helps explain inter-individual variability in marginal bone stability.	Biomarker-driven patient stratification studies are lacking.
Translational proof of axis relevance	Experimental and clinical anti-sclerostin studies indicate that adult cortical bone can regain anabolic activity when inhibitory signaling is reduced.	Indirect; not implant-specific and not proposed as a local clinical intervention here.	Supports the plausibility that cortical apposition can be enhanced if the inhibitory axis is transiently reduced.	Systemic pharmacological evidence cannot be extrapolated directly to localized peri-implant modulation.

**Table 2:** Candidate non-pharmacological directions relevant to the proposed osteocyte axis in peri-implant bone.

Candidate Non-Pharmacological Direction	Proposed Interaction with the Osteocyte Axis	Expected Structural Consequence if Successful	Evidence Status	Main Caveat
Controlled dynamic loading or staged loading protocols	Provide cyclic mechanical input more likely to suppress SOST and maintain Wnt-mediated anabolic signaling than static or monotonous loading.	Greater probability of surface apposition and cortical reinforcement rather than turnover alone.	Indirect mechanistic rationale with implant-related biomechanical support.	Poorly controlled timing or excessive magnitude may instead intensify remodeling or microdamage.
Load-distribution optimization through implant or prosthetic design	Reduces adverse crestal stress concentration and shapes the local mechanical signal profile reaching cortical osteocytes.	More favorable conditions for transition from remodeling-dominant to modeling-dominant behavior.	Moderate biomechanical relevance; limited direct osteocyte evidence.	Design effects are multifactorial and may be confounded by surgical and host factors.
Microenvironment stabilization during early healing	Improved perfusion and inflammatory resolution may lower the threshold for Wnt-mediated osteogenesis and reduce persistence of a SOST-high state.	More stable marginal adaptation and improved cortical maturation.	Biologically plausible; direct peri-implant SOST data remain limited.	Difficult to isolate microenvironmental effects from general wound-healing quality.
Biomaterial-assisted local healing support	Local matrices or surfaces that support vascularization and early healing may indirectly bias the osteocyte environment toward anabolic signaling.	Enhanced cortical maturation if coupled with favorable mechanical conditions.	Conceptual to early translational stage.	Effects may reflect general osteoconduction rather than selective osteocyte modulation.
Localized biophysical adjuncts	Non-systemic physical stimulation may transiently modulate mechanotransduction and the SOST/Wnt balance during the healing window.	Potential support for net cortical apposition without systemic exposure.	Hypothesis-generating; requires targeted validation.	Selectivity, dosing, timing, and reproducibility remain unresolved.
Patient-specific protocol adjustment	Protocols tailored to age, cortical phenotype, or systemic bone risk may better match the osteocyte activation threshold of a given patient.	Improved predictability of marginal bone stability across heterogeneous hosts.	Indirect but clinically relevant.	Requires reliable markers for biological stratification.

**Table 3:** Testable predictions and translational endpoints emerging from the proposed framework. Feasibility windows are illustrative and intended to guide study planning; they do not substitute for formal sample-size estimation or power analysis.

Framework-Derived Prediction	Suggested Study Model	Molecular Endpoints	Structural or Clinical Endpoints	Interpretation if Confirmed	Illustrative Feasibility Window (Study Duration/Scale; Not Power-Based)
Dynamic loading with appropriate amplitude and frequency will be associated with lower SOST expression and greater Wnt-related anabolic signaling than static or monotonous loading.	Animal implant model or controlled ex vivo loading model.	SOST/sclerostin, β-catenin activity, RUNX2, ALP, OCN, RANKL/OPG.	Cortical thickness, bone-to-implant contact, peri-implant bone volume, resonance frequency analysis.	Supports the role of mechanical signal profile in shifting the osteocyte regulatory threshold.	Pilot preclinical implant study; 4–8 weeks after implantation, preferably with unloaded, static, and dynamic comparator groups plus serial early readouts.
Interventions that improve local perfusion and inflammatory resolution will be associated with a lower probability of persistent SOST-high remodeling-dominant behavior.	Preclinical healing model with longitudinal sampling.	SOST/sclerostin, VEGF, HIF-related markers, IL-1β, TNF-α, OPG/RANKL.	Marginal bone level, histomorphometry, cortical maturation, implant stability.	Supports the microenvironmental arm of the framework.	Preclinical longitudinal healing study; serial sampling across the first 1–6 weeks, with terminal structural assessment by approximately 6–8 weeks.
Host conditions associated with reduced mechanosensitivity will show a higher threshold for transition to modeling-dominant apposition.	Stratified animal or clinical cohort study.	SOST/sclerostin, Wnt-related markers, serum bone turnover markers.	Marginal bone change, cortical thickness, implant survival, implant stability.	Supports patient-specific variability in osteocyte responsiveness.	Stratified animal study or observational clinical cohort; approximately 8–12 weeks preclinically or at least 12 months clinically, with predefined host-risk strata.
An increase in turnover without a parallel reduction in SOST will not be sufficient to produce durable cortical thickening.	Experimental comparison of trauma-based stimulation versus controlled anabolic conditions.	SOST/sclerostin, TRAP, cathepsin K, ALP, OCN.	Net cortical apposition, bone-to-implant contact, crestal stability.	Distinguishes remodeling intensification from true modeling-driven reinforcement.	Mechanistic preclinical comparison; 4–8 weeks, using matched trauma-dominant versus anabolic conditions with terminal histology and micro-CT.
The early healing window will be more responsive to local modulation of the osteocyte axis than later phases after regulatory setpoints have stabilized.	Time-course implant healing study.	Serial SOST/sclerostin and Wnt-related markers.	Time-dependent changes in cortical thickness and marginal bone levels.	Identifies the most relevant translational intervention window.	Time-course study focused on early healing; serial assessments at days 3–7 and weeks 2, 4, and 8, with optional clinical follow-up at 3, 6, and 12 months.
Isolated molecular change without structural gain will be insufficient to validate the framework clinically.	Translational study combining biomarkers with imaging and biomechanics.	Local and systemic biomarkers.	CBCT or micro-CT, histology, resonance frequency analysis, marginal bone change.	Reinforces the need for combined biological and structural endpoints.	Pilot translational cohort or interventional study; minimum 12-month follow-up, ideally 12–24 months, combining biomarkers, imaging, and biomechanical testing.

## Data Availability

No new data were created or analyzed in this study. Data sharing is not applicable to this article.
